# Older and younger adults differ in time course of skill acquisition but not in overall improvement in a bimanual visuomotor tracking task

**DOI:** 10.3389/fnagi.2024.1373252

**Published:** 2024-04-11

**Authors:** Ana Zvornik, Keenie Ayla Andersen, Andreas Deigaard Petersen, Mikael Novén, Hartwig Roman Siebner, Jesper Lundbye-Jensen, Anke Ninija Karabanov

**Affiliations:** ^1^Department of Nutrition, Exercise, and Sports, Faculty of Science, University of Copenhagen, Copenhagen, Denmark; ^2^Danish Research Centre for Magnetic Resonance, Centre for Functional and Diagnostic Imaging and Research, Copenhagen University Hospital Amager and Hvidovre, Hvidovre, Denmark; ^3^Department of Neurology, Copenhagen University Hospital Bispebjerg and Frederiksberg, Copenhagen, Denmark; ^4^Institute for Clinical Medicine, Faculty of Medical and Health Sciences, University of Copenhagen, Copenhagen, Denmark

**Keywords:** motor learning, visual tracking, bimanual actions, aging, skill, visuomotor ability

## Abstract

Manual motor performance declines with age, but the extent to which age influences the acquisition of new skills remains a topic of debate. Here, we examined whether older healthy adults show less training-dependent performance improvements during a single session of a bimanual pinch task than younger adults. We also explored whether physical and cognitive factors, such as grip strength or motor-cognitive ability, are associated with performance improvements. Healthy younger (*n* = 16) and older (*n* = 20) adults performed three training blocks separated by short breaks. Participants were tasked with producing visually instructed changes in pinch force using their right and left thumb and index fingers. Task complexity was varied by shifting between bimanual mirror-symmetric and inverse-asymmetric changes in pinch force. Older adults generally displayed higher visuomotor force tracking errors during the more complex inverse-asymmetric task compared to younger adults. Both groups showed a comparable net decrease in visuomotor force tracking error over the entire session, but their improvement trajectories differed. Young adults showed enhanced visuomotor tracking error only in the first block, while older adults exhibited a more gradual improvement over the three training blocks. Furthermore, grip strength and performance on a motor-cognitive test battery scaled positively with individual performance improvements during the first block in both age groups. Together, the results show subtle age-dependent differences in the rate of bimanual visuomotor skill acquisition, while overall short-term learning ability is maintained.

## Introduction

Manual motor control worsens during healthy aging. Adults above the age of 60 years consistently show increased error rates, higher variability, and greater movement execution times during bimanual coordination tasks when compared to their younger counterparts (Vieluf et al., 2015; Rudisch et al., 2020; Danuta and Tokarski, 2021; Kang et al., 2022). The age-dependent decrease in manual performance is not unique to bimanual skills (Vieluf et al., 2012; Vasylenko et al., 2018) but there is a tendency for more pronounced deficits during asymmetrical bimanual tasks when compared to simpler manual movements (Kang et al., 2022). The ability to maintain good bimanual performance across the lifespan is crucial for upholding an independent lifestyle in older age, enabling basic activities of daily living, such as buttoning a shirt or opening a container. Older adults also need to maintain the ability to acquire new bimanual skills as smartphones, tablets, and controllers for virtual environments continuously extend the set of relevant motor skills that may be mastered.

While the negative impact of age on manual control is well documented, the evidence that the learning rates are affected by age is more inconclusive. While some single-session motor learning studies have found reduced learning rates in older adults (Etnier and Landers, 1998; Hegele and Heuer, 2013) others report preserved or even increased learning rates in older age (Bhakuni and Mutha, 2015; Vieluf et al., 2015; Berghuis et al., 2019). A common factor in studies finding an age-dependent decline in improvement rate is task complexity. While rates for low-complexity tasks are very similar across age groups, more complex tasks often reveal differences between older and younger adults (Voelcker-Rehage, 2008). During bimanual tasks, complexity is partially determined by the bimanual context. Symmetric movements, which require both hands to move in an identical fashion, are less complex than asymmetric movements where the hands perform different actions. Generally, older adults show more deficits when bimanual actions require asymmetric movements than when the arms or hands move symmetrically (Bangert et al., 2010; Kang et al., 2022), but it is not clear whether that means that learning rates for asymmetric bimanual movements are more impaired in older adults than learning rates for symmetric bimanual movements.

Studies that have investigated age-dependent differences in motor learning often quantify improvements by calculating gain scores (e.g., difference between start and end performance) but assessing the entire time course of motor-skill learning over individual trials gives a more nuanced image of improvement (Anderson et al., 2021). Focusing on the entire time course of early skill learning instead of average gain scores, Bastrop et al. have demonstrated that performance improvements in healthy young adults seem to occur during short periods of rest, rather than during continuous practice within a training block (Bönstrup et al., 2019, 2020). The observed improvement gains during breaks between training blocks have been interpreted as a rapid form of consolidation that substantially contributes to early skill learning and it is unclear whether similar “micro-offline” consolidation can also be observed in motor tasks with a different temporal structure (e.g., fewer breaks or longer blocks) and if the learning trajectory within and between blocks is different between younger and older adults. Gaining insights on age-dependent differences in learning trajectories is important for developing evidence-based recommendations for training new fine motor skills at different ages.

Previous studies have also investigated which factors may contribute to individual differences in manual motor performance and motor learning (Liu et al., 2017). Manual motor performance and motor learning correlate with hand grip strength and physical fitness (Etnier and Landers, 1998; Etnier et al., 2001; Martin et al., 2015; Hubner and Voelcker-Rehage, 2017; Liu et al., 2017; Hübner et al., 2019). However, studies do not agree on the direction of the association: while some studies report a positive association between cardiovascular fitness and the acquisition of visuomotor tasks (Etnier and Landers, 1998; Etnier et al., 2001) others report negative associations between acquisition rates and physical fitness as well as grip strength (Hübner et al., 2019). The unexpected negative associations may, however, be mediated via the positive effect of fitness and grip strength on initial performance and the fact that participants with better initial performance showed lower learning rates (Hübner et al., 2019). In addition to physical factors, cognitive performance has been associated with fine motor coordination in older adults (Niemann et al., 2016), but usually, studies do not attempt to discern the individual contribution of motor and cognitive factors included in a combined regression model. Hence, we recorded measures of grip strength, physical fitness, and cognitive function in all our participants to explore their individual association motor performance and learning rate when simultaneously controlling for the other factors.

We have recently introduced a bimanual pinch force task that allows us to study visually cued shifts in coordination contexts (Karabanov et al., 2023). One of the advantages of the paradigm is that it allows a dynamic succession between simpler symmetric and more complex asymmetric movements that resemble tasks like using a controller to a high degree. In the present study, we used this experimental setup to investigate the effect of simple and complex bimanual coordination tasks (e.g., inverse-asymmetric vs. mirror-symmetric coordination) on age-dependent training improvements during a single training session, consisting of 3 11.5 min blocks with short between-block breaks. We hypothesized that older adults would show a greater increase in error when shifting from simple mirror-symmetric to the complex, inverse-asymmetric coordination context than younger adults (Rudisch et al., 2020). Regarding learning, we expected to see higher improvement rates for the younger participants. Additionally, we performed exploratory correlational analysis among grip strength, manual dexterity, physical fitness and cognitive functions, and baseline performance and learning rates in the visuomotor tracking task.

## Methods

### Participants

In total, 36 healthy participants with normal or corrected-to-normal vision were included in the study. The participants were recruited by online advertisements and flyers. In total, 16 of them were assigned to a subsample of younger adults in the age range of 18–35 years (25.9 ± 3 years; 9 women) while 20 were assigned to a subsample of older adults in the age range of 60–80 years (68.5 ± 3.5; 13 women). All participants were right-handed as confirmed by the Edinburgh Handedness Inventory (Oldfield, 1971). None of the participants reported a personal history of psychiatric, neurological, or cardiovascular illness or injury or the current intake of neuroactive medications. All participants were screened for cognitive impairment using the Montreal Cognitive Assessment tool (MoCa) (Nasreddine et al., 2005) (average score young: 28.3-point average ± 2.6; old: 28.1-point average ± 1.6). All participants received oral and written information about the study and gave written, informed consent before participation The study was approved by the Regional Committee on Health Research Ethics of the Capital Region in Denmark (De Videnskabsetiske Komitéer-Region H; Journal-nr: H-21025010).

### Experimental procedure

**Figure 1:**
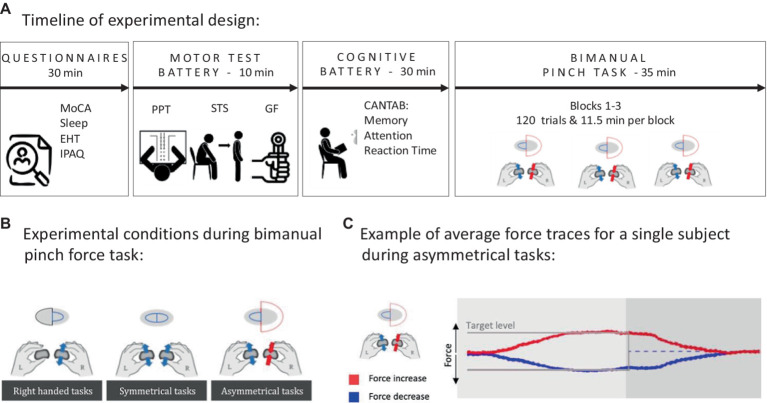
Illustration of experimental design. **(A)** An overview of the experimental flow as shown in the diagram. Questionnaires: Participants completed the Montreal Cognitive Assessment (MoCA), Edinburgh Handedness Test (EHT), and International Physical Activity Questionnaire (IPAQ) and gave information about their sleep duration and quality the previous night. The motor test battery included the Purdue Pegboard Test (PPT), a 30 s sit-to-stand test (STS), and a hand grip force assessment (GF). The Cognitive test battery used the Cambridge Neuropsychological Test Automated Battery (CANTAB) and tested memory (paired associative learning), attention (rapid visual information), and reaction time (RT). Finally, all participants completed three blocks of the bimanual pinch task. Rest intervals between the individual sections were self-paced by the participant, but never exceeded 5 min. **(B)** Three different conditions during the bimanual pinch force task where pinch movements either could be performed only with the right hand, mirror symmetrically with both hands, and inverse-asymmetrically with both hands. **(C)** The average force traces of a single subject during the inverse-asymmetrical condition.

At the start of the experiment, the participants received general information about the experiments and signed the consent form. Participants also filled out questionnaires assessing handedness [Edinburgh Handedness Inventory [EHI] (Oldfield, 1971)] and physical activity levels (International Physical Activity Questionnaire [IPAQ]) (Craig et al., 2003). The (MoCa) (Nasreddine et al., 2005) screened for signs of mild cognitive impairment. After completing the questionnaires, participants’ manual function was assessed for unimanual and bimanual dexterity [Purdue Pegboard Test [PPT] (Tiffin and Asher, 1948)] and grip strength in both hands (Baseline^®^ Hydraulic Hand Dynamometers). Additionally, a 30 s sit-to-stand test (Lein et al., 2022) tested lower extremity strength and served as an approximator for submaximal fitness. After the physical tests, participants also completed three sub-tests of the Cambridge Neuropsychological Test Automated Battery (paired associative learning [PAL], rapid visual information [RVI], and reaction time [RT]; CANTAB^®^, Cambridge Cognition 2019, www.cantab.com) to assess working memory, attention, and reaction time. Finally, the participants completed three blocks of the visually guided bimanual pinch-grip task described below. Including breaks, the bimanual pinch-grip task lasted approximately 35 min (see Figure 1A) during which EEG was recorded using a 64-channel BioSemi system (BioSemi Inc., Amsterdam, Netherlands). The EEG data are going to be reported elsewhere.

### Bimanual grip task

Two small, round, custom-built force transducers (25.4 mm in diameter) were used to assess force during the visually cued bimanual pinch-grip task. Participants were holding a transducer in each hand using a precision “key grip” where the pad of the thumb and the side of the index finger had contact with the force sensor (Palastanga et al., 1994). By increasing or decreasing the pinch force in each hand, participants could control the size of two visually presented semi-circles on a screen: the right semi-circle was controlled by the right hand, and the left semi-circle was controlled by the left (see Figures 1B,C for an example of the task). The sampling rate of the force sensor was 1,024 Hz, and the visual signal displayed on the screen was smoothed by averaging over the last 100 samples to minimize electrical background noise. For collecting the force data and providing visual feedback to the participants, a customized PsychoPy script was used.

The experimental task required the participants to match their pinch force with the target as precisely as possible. The target consisted of two adjacent blue semi-circles, their size indicating the target force for the left and right hand, respectively. The pinch force of the participants was visualized by overlaid yellow semi-circles; their size was controlled by the pinch force of the participants: increasing force increased the semi-circle size while decreasing force decreased their size. Both semi-circles representing the target force level and semi-circles representing the actual force produced by the participants were displayed in the middle of a 24″ monitor placed approximately 70 cm in front of the participants. The individual maximal voluntary contraction (MVC) of each hand was used to set the target force to one of three levels: baseline (5% MVC), force increase (7.5% MVC), and force decrease (2.5% MVC). The experiment began with both hands at a tonic contraction at the baseline level. Each trial (total duration of 4 s) consisted of a 2 s change in target force to either 2.5% or 7.5% MVC, a 2 s return to baseline (5% MVC), and a jitter time of ±0.1 s was added to each 2 s interval. This setup allowed contrasting bimanual coordination patterns where both hands pressed mirror-symmetrically (SYM) with pinch force changing in the same direction and coordination patterns where both hands pressed inverse-asymmetrically (ASYM) with pinch force changing in opposite directions. Finally, the setup also included pseudo-unimanual movements (UNI) where only the right hand changed pinch force while the left remained at baseline; however, in this study, we are focusing on contrasting the bimanual conditions. In total, 120 trials were recorded for each condition (120 SYM consisting of 60 *decrease* and 60 *increase* trials; 120 ASYM consisting of 60 right *decrease*/left *increase* and 60 right *increase*/left *decrease* trials; 120 UNI consisting of 60 right *decrease* and 60 right *increase* trials). Additionally, 60 baseline trials were added where the force remained stable through the entire 4 s trial. Hence, a total number of 420 trials were recorded from each participant. Trials were separated into three 11.5 min blocks with 120 trials per block to allow for short breaks during the task. The duration of the break between blocks was determined by the participant but never exceeded 5 min.

### Outcome variables

The primary outcome variables of quantity performance during the task were error and reaction time (RT). To calculate these measures, the smoothed force data used of the left and right hands was extracted for all individual trials. Error was calculated as the area between the target trace and the force trace during the last 500 ms of the 2 s periods where participants had to hold the pinch force at the indicated target level. This time interval was chosen to ensure that participants had reached a steady force. RT was calculated on averaged force traces for each participant, block, and condition and determined as the earliest timepoint after the pinch force deviated more than 15% from the last 100 ms. We decided to calculate RT on the average traces as no reliable estimate of RT could be established using the individual force traces. We focused on SYM and ASYM when defining bimanual context as UNI movements involved a tonic contraction in the left hand while the right hand changed force level between hands; hence, they can be seen as a “simpler” asymmetric movement.

Secondary outcome measures included motor and cognitive tests: the motor-cognitive assessment served two purposes: first as a thorough characterization of the two experimental groups and second to allow further exploratory analysis correlating bimanual visuomotor performance and learning with indicators of basic cognitive-motor control. To limit the number of statistical tests, we restricted the number of cognitive–motor variables to core measures of (1) grip strength (averaged across left and right hands), (2) bimanual dexterity (assembly task of PPT), (3) estimated submaximal fitness (sit-to-stand), and (4) a composite cognitive score, calculated as the standard score of PAL, RVI, and RTI. To characterize performance on PAL, RVI, and RTI, we identified 1–2 measures of each test that we thought best represented average performance (PAL: total error-stage-6; RVI: %correct and mean latency; RTI: mean response time).

### Statistics

Statistical analysis of all data was performed using the R 3.6.0 base package RStudio (version 2022.07.2; build 576) R Core Team (2019). To test for the effects of age group (old vs. young) and bimanual context (SYM vs. ASYM) on bimanual learning, a linear mixed model was calculated using the R-package lme4 (Bates et al., 2015). Significant main effects or interactions were evaluated using the R-package lmerTest, which computes *p*-values from mixed-effects models using Satterthwaite’s degrees of freedom approach (Kuznetsova et al., 2017). If significant main effects or interactions were observed, pairwise comparisons were made using the *emmeans* R-package. For error, the effects of within-block improvements (trials; 1–20) and between-block improvements (block; 1–3) were modeled separately as the error could be extracted on a trial-to-trial basis. For RT, the variable trial was dropped from the model as RT could only be calculated on the averaged traces across all trials of one condition within a block.

For variables where a significant group-by-training interaction was observed, we also calculated the overall normalized learning gain to separate differences in learning slope from overall improvements during training. The overall gain, g, was calculated using the following formula: (Initial Error − Final Error)/Initial Error (Hake, 1998).

For all models, the normality of the residuals was checked using the ggplot function and all dependent variables were log-transformed to ensure the normality of the residuals. *Post-hoc* analysis of significant main effects and interactions was performed using Tukey’s honest significant difference. To detect group differences in the cognitive–motor variables, independent *t*-tests were used. For variables where a significant group-by-training interaction could be observed, the normalized learning gain, g, was compared between groups using an independent *t*-test to separate differences in learning slope from the overall gain across the entire session.

Two linear models were constructed for the exploratory correlation analysis: first focusing on early learning with learning gain g over the first block (error − early learning) as the outcome variable and another one focusing on learning over the entire block with learning gain g over the entire experiment (error − total learning) as the outcome variable. For both models, the cognitive–motor variables and the initial performance were used as continuous predictors, and the groups were used as categorical predictors of g. The linear models were calculated using the lm function published in the R stats package (Team, 2018). The threshold for null hypothesis testing was set to *p* < 0.05 for all statistical tests.

## Results

### Bimanual pinch force task – error

**Figure 2:**
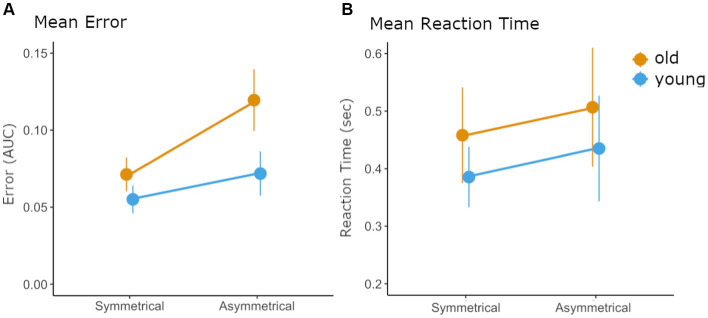
Mean error and reaction time values for each group and each condition. **(A)** Significant task-by-group interaction for the error data indicating that the error of older participants increases more in the asymmetrical task than the error of younger participants. Error is expressed as the area under the curve (AUC) between the target level and force trace for the last 500 ms. **(B)** The main effect of task and group for the reaction time data indicating that reaction time is slower for older participants and that both groups show similar slowing of reaction time during the asymmetrical task. Both graphs show standard error by vertical lines.

For error, all main effects were significant: older participants had significantly higher error values than younger (*F* (1) = 13.07; *p* = 0.001), ASY trials resulted in significantly higher error than SYM (*F* (1) = 566.808; *p* < 0.001), and error generally decreased over the three blocks (*F* (2) = 102.69; *p* < 0.001) and individual trials within a block (*F* (1) = 17.46; *p* < 0.001). Significant interactions were also observed: Age interacted with the bimanual context (*F* (1) = 78.79; *p* < 0.001) (Figure 2A). For this interaction, the *post-hoc* tests indicated that during ASY trials the older participants’ error was significantly greater than that of the young participants but that this was not the case for SYM trials (ASY-young vs. ASY-old: *p* < 0.001; SYM-young vs. SYM-old: *p* = 0.08).

**Figure 3:**
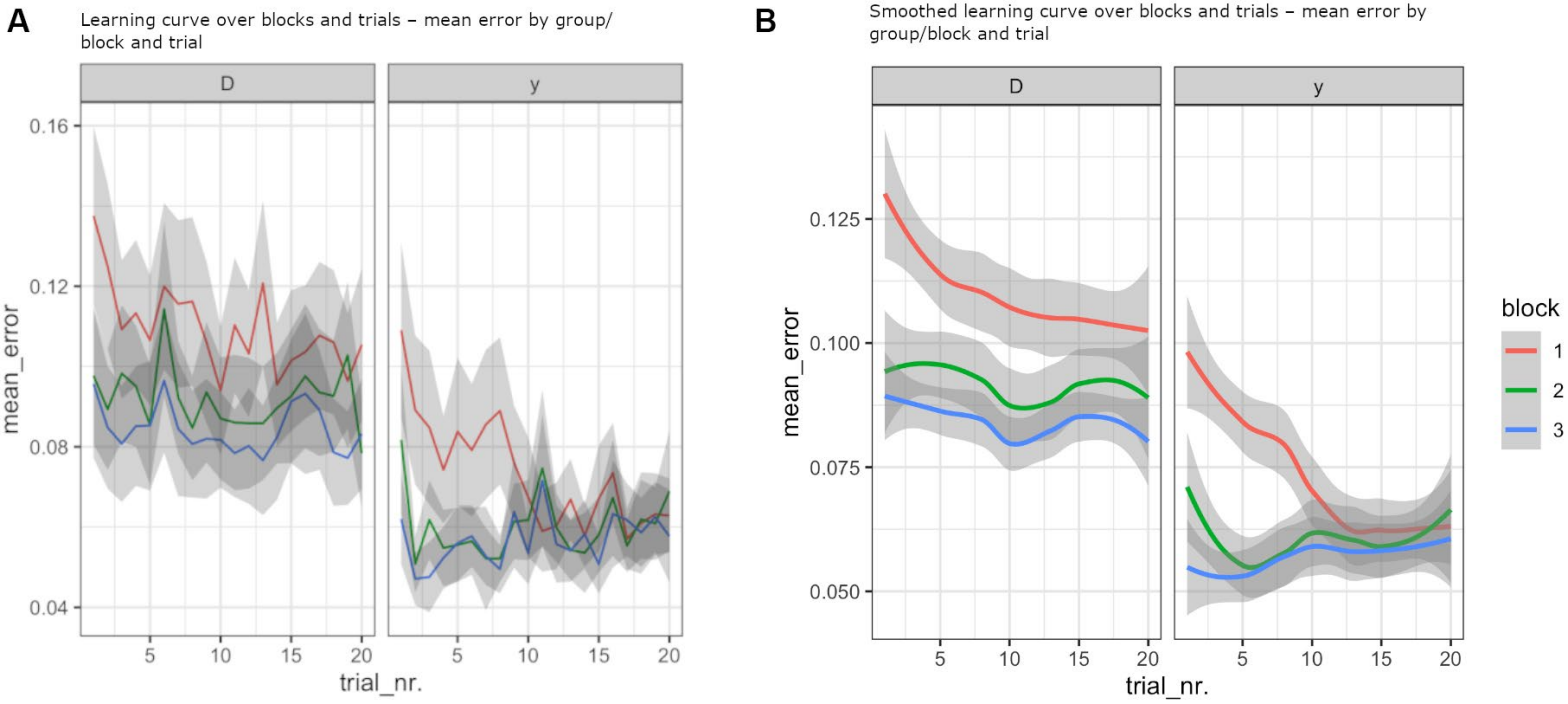
Older adults showed different within and between-block learning slopes when compared to young adults. **(A)** Mean error over trials for each group (old to the left and young to the right) and for each block (red, green, and blue lines). The standard error is depicted in grey. **(B)** Smoothed conditional means. In all graphs, error is expressed as the area under the curve (AUC) between the target level and force trace for the last 500 ms.

Moreover, there was a significant three-way interaction: age interacted with block and trial number (*F* (2) = 5.26; *p* = 0.005), indicating that older adults showed different within- and between-block learning slope when compared to young adults (Figure 3). *Post-hoc* tests between the first and the last trials of each block confirmed that younger adults only showed significant improvement during the first block (Block1Trial1 vs. Block1Trial20, *p* < 0.001). Older participants also showed improvement during the first block (Block1Trial1 vs. Block1Trial20, *p* < 0.001) but additionally improved between the blocks (Block1Trial20 vs. Block3Trial20, *p* < 0.001 and Block2Trial1 vs. Block3Trial20, *p* < 0.01) (Figure 3). While the three-way interaction indicated that the learning slopes were different for the older and younger participants the overall gain g over the entire experiment was not significantly different between the groups (*p* > 0.5; independent *t*-test). Mean values for each group, condition, and block can be seen in Table 1.

**Table 1:** Mean error values for each group, condition, and block.

Error	Symmetry			Asymmetry		
	B1	B2	B3	B1	B2	B3
Old	0.08 (0.02)	0.06 (0.015)	0.06 (0.011)	0.14 (0.032)	0.11 (0.027)	0.10 (0.025)
Young	0.06 (0.009)	0.05 (0.01)	0.05 (0.008)	0.08 (0.017)	0.07 (0.011)	0.06 (0.008)

Standard derivation in brackets.

### Bimanual pinch force task – reaction time

For RT all main effects were significant: The effect for group (*F* = 12.0; *p* = 0.001) indicated that older participants exhibited significantly longer RT than the young participants. The significant main effect of bimanual context (*F* = 28.6; *p* < 0.001) indicated that both groups experienced longer RT during ASY tasks (Figure 2B) and the main effect of the block (*F* = 7.6; *p* = 0.006) indicated that all participants decreased their reaction time over the three blocks. *Post-hoc* tests on the effect of block revealed that RT in block 1 was significantly higher than RT in block 3 (*p* = 0.004). No interaction between factors was significant (all *p*-values >0.7037). Mean values for each group, condition, and block can be seen in Table 2.

**Table 2:** Mean reaction time (RT) values for each group, condition, and block.

RT	Symmetry			Asymmetry		
	B1	B2	B3	B1	B2	B3
Old	0.48 (0.08)	0.45 (0.08)	0.44 (0.08)	0.50 (0.11)	0.52 (0.09)	0.49 (0.09)
Young	0.39 (0.05)	0.38 (0.05)	0.37 (0.04)	0.44 (0.09)	0.43 (0.09)	0.42 (0.08)

Standard derivation in brackets.

### Motor-cognitive test battery

**Figure 4:**
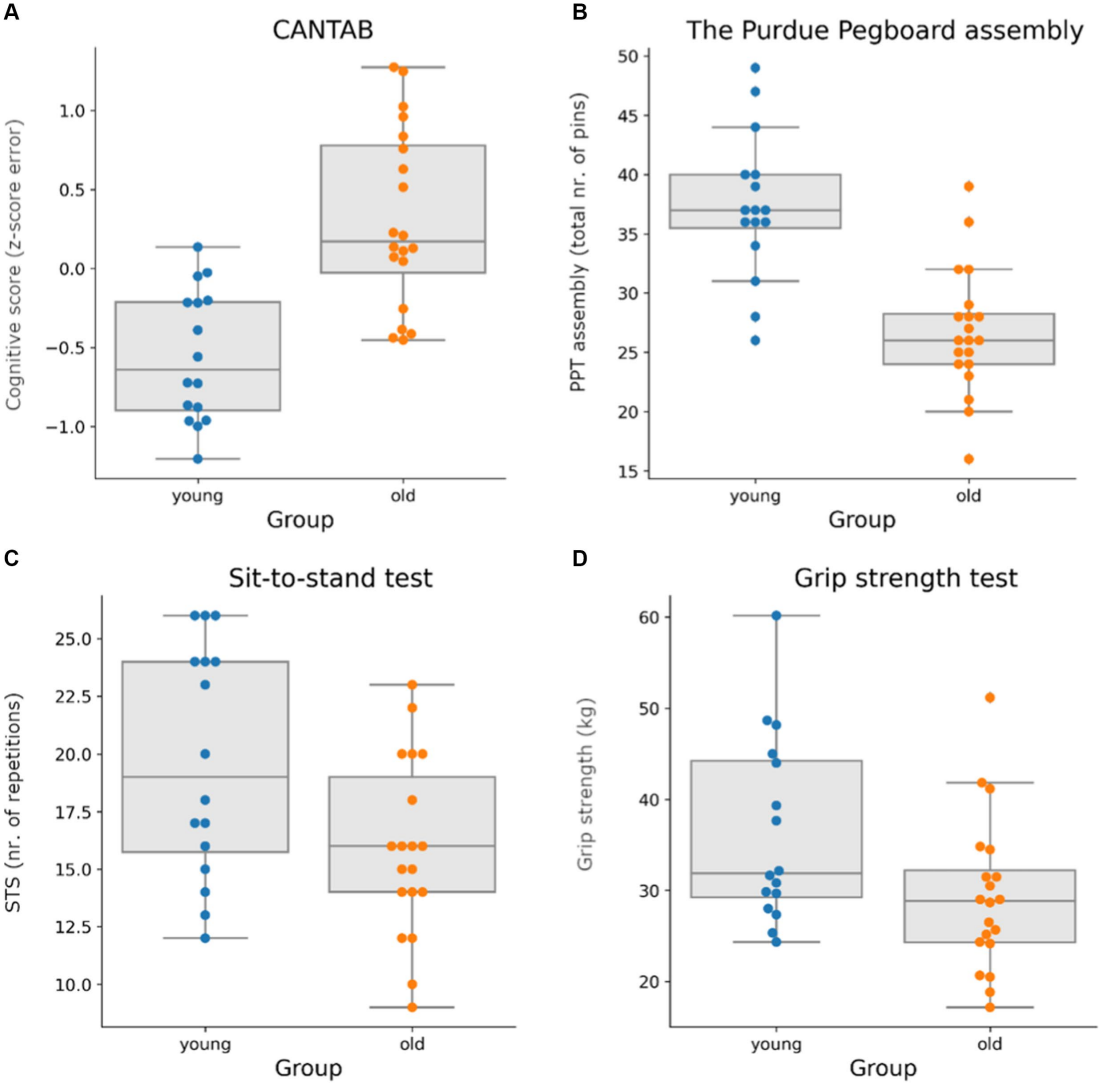
Comparison of the results of the cognitive and motor test battery shows that the younger group (blue) outperforms the older group (orange) in all tests. **(A)** Composite *z*-score of cognitive tasks performed on CANTAB. The tasks included are rapid visual information processing, paired associative learning, and reaction time, note that the composite score is predominantly error-based; hence, a low score indicates good performance. **(B)** Results of the assembly task for the Purdue Pegboard (PPT). The *y*-axis denotes the total number of assembled items; hence, a high number indicates good performance. **(C)** Results of the sit-to-stand test (STS). The *y*-axis denotes the number of repetitions performed during 30 s; hence, a high number indicates good performance. **(D)** Results of the grip strength task, averaged across the left and right hands. The *y*-axis denotes strength in kilograms.

The results of the IPAQ suggested that groups were comparable regarding their level of physical activity as the metabolic equivalent (MET)-minutes per week showed no significant difference between older and younger participants (*p* > 0.5; mean older = 1985 MET min/week; mean younger = 2,397 MET min/week). Despite the similarity in physical activity, the study revealed statistically significant differences in all motor and cognitive tests between the young and older groups. In the sit-to-stand task, the younger group achieved a significantly higher number of repetitions than their older counterparts (Figure 4C, *t* = −2.53; *p* = 0.016). For grip strength, younger participants displayed significantly higher values than the older group (Figure 4D, *t* = −2.36; *p* = 0.030). In addition, in the pegboard test, the younger group could perform a significantly higher number of assemblies than the older group (Figure 4B, *T* = −5,5,486; *p* < 0.001). Regarding Cantab *z*-score the older group had a significantly higher composite score than the young group (Figure 4A, *t* = 5.10; *p* < 0.001), indicating that they made more errors in the cognitive tests than the young group.

### Correlations

**Figure 5:**
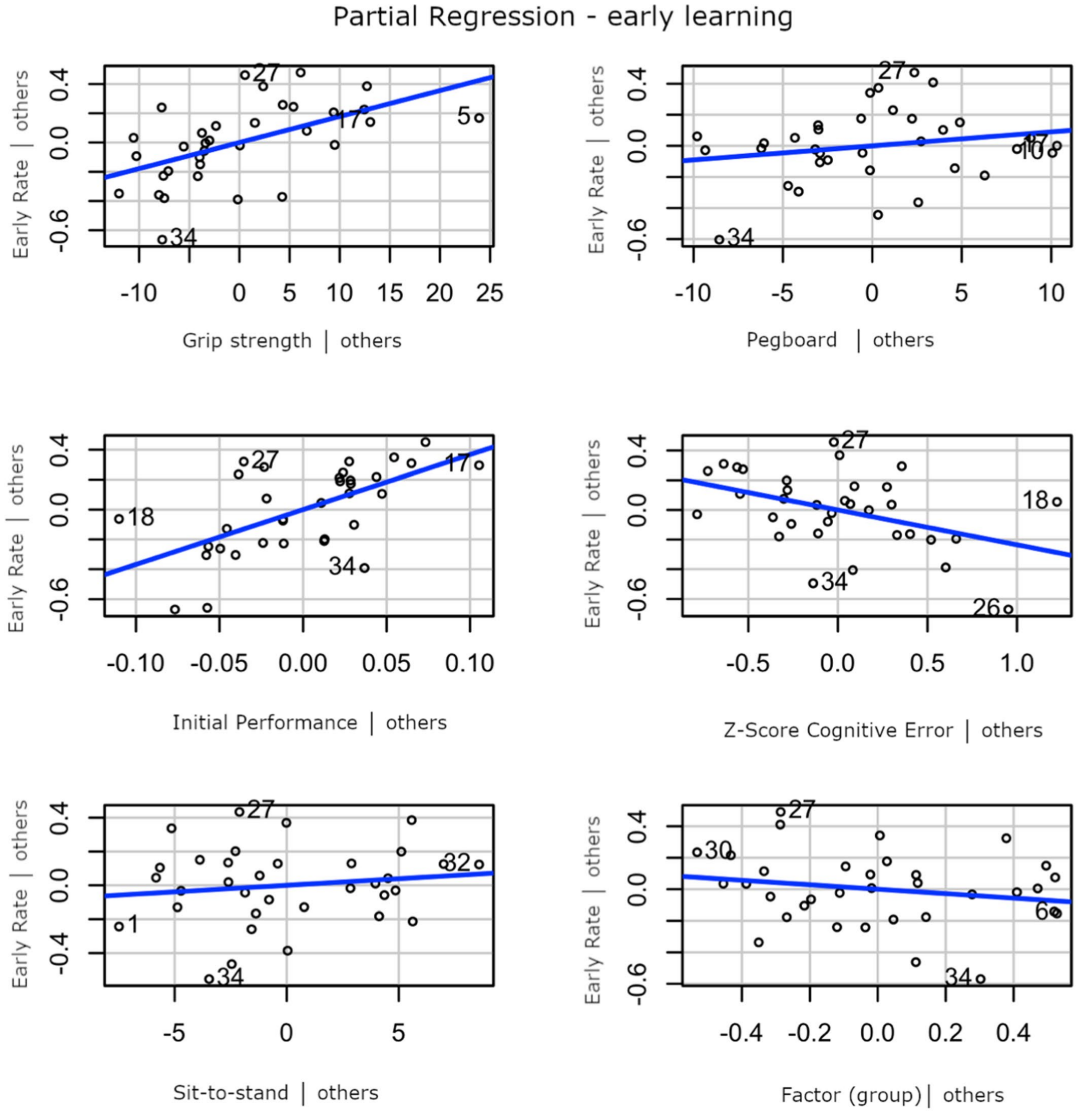
Partial correlation between early learning rate and grip strength (top left), pegboard assemblies (top right), initial performance (middle left), Cantab *z*-score (middle right), sit-to-stand test (lower left), and group (lower right).

The linear model that modeled early learning as a function of pegboard assemblies, grip strength, Cantab *z*-score, sit to stand, and initial performance as continuous predictors and the group as a discrete factor explained a significant proportion of variance (*p* < 0.001; *F* (6) = 7.00; *R*^2^ = 0.60; *R*^2^-adjusted = 0.52) with higher performance on grip strength and the Cantab *z*-score predicting higher early learning rates. In addition, a high initial error was associated with a high learning rate. When looking at the importance of individual predictors, grip strength, Cantab *z*-score, and initial performance contributed significantly to early learning (*p* = 0.002 and *p* = 0.004 and *p* = 0.01, respectively), while group and pegboard did not have a significant predictive value (*p* > 0.2). The partial correlations between individual factors and early learning rate are shown in Figure 5. The linear model that used the same predictors to predict the total learning rate over all three blocks explained a significant proportion of variance (*p* = 0.007; *F* (6) = 3.75; *R*^2^ = 0.45; *R*^2^-adjusted = 0.33). When looking at the importance of individual predictors, only initial performance had a significant effect on the final learning rate (*p* < 0.001).

## Discussion

We found age-related differences in bimanual performance and learning. Compared to symmetric pinch force tracking, increased complexity during asymmetric pinch force tracking had a more pronounced detrimental effect on visuomotor tracking error in older adults. Older participants also exhibited nuanced distinctions in learning trajectories across the three blocks in comparison with their younger counterparts. Older adults also showed a general slowing of reaction time but without a discernible group effect of movement context or learning on reaction time.

### Age-dependent effect on the time course of skill acquisition

While the net learning gain over the entire training session was the same for younger and older adults, the two groups showed differences in the rate of learning across the three blocks. Younger adults showed a faster reduction in force tracking error during the first 15 repetitions and plateaued after that. Older adults improved more continuously over the training session and were able to significantly improve their performance after the first block. This observation highlights the need for analyzing the entire time course of motor-skill acquisitions, instead of averaged gain scores in order to understand the divergent results concerning age-dependent differences in manual skill learning in the previous literature (Etnier et al., 2001; Hegele and Heuer, 2013; Bhakuni and Mutha, 2015; Vieluf et al., 2015; Berghuis et al., 2019). Average scores may yield quite different results depending on where in the learning process averages are extracted and considering the entire time course of acquisition will help to evaluate if both groups have reached a comparable learning stage, as indicated by a comparable plateauing of improvement.

The finding that age specifically affects early learning rates in visuomotor learning is in line with several studies looking at learning rates during visuomotor adaptation (Vachon et al., 2020; Wang et al., 2022; Ruitenberg et al., 2023). These studies observed that the early adaptation phase, primarily driven by cognitive processes, is slower in older adults while later, implicit adaptation processes are spared. Previous studies have shown stronger effects of aging on cerebellar-mediated motor learning forms such as motor adaptation than on other classical learning paradigms such as motor sequence learning (Seidler, 2006). While motor adaptation and sequence learning are the most common paradigms of motor learning, they are not a good model for the learning of novel visuomotor mappings that characterize the acquisition of many motor skills (Krakauer et al., 2019). Variations of visuomotor tracking tasks such as the task used here may be better at testing for *de novo* learning of arbitrary visuomotor association and involve the cerebellum as well as the parietal and premotor cortex. While there is mixed evidence for age-dependent effects on visuomotor tracking in general (Etnier and Landers, 1998; Berghuis et al., 2019) we are not aware of other studies that have investigated differences in the time course of skill acquisition in this type of motor learning tasks. Our results suggest that age-dependent differences in skill acquisition may be most prominent during the very early phase of forming de-novo sensorimotor associations, thereby mirroring the age-dependent learning effects observed during visuomotor adaptation.

### Age-dependent effects of task complexity

Older adults were more challenged by the increasing complexity of bimanual coordination, showing higher pinch force tracking error scores during inverse-asymmetric movements, compared to younger adults. In contrast, pinch force tracking errors did not significantly differ between groups during mirror-symmetric movements. This finding supports previous studies reporting that age-related performance differences increase with task complexity (Smith et al., 1999; Bangert et al., 2010; Bootsma et al., 2021; Van Ruitenbeek et al., 2023) and that older adults are more affected by increasing complexity in motor tasks (Rudisch et al., 2020; Kang et al., 2022). This is not unique to motor learning as a general tendency for older adults to be more sensitive to increases in task difficulty has also been reported in other task domains (Zhang et al., 2019). It is worth noting that we cannot determine whether the difference in complex bimanual coordination arises from the need to coordinate a more complex motor program, from age-dependent effects on visual information processing (Guest et al., 2015) or a combination of both factors. However, studies investigating the neural mechanisms that underlie the age-dependent decline suggest that fontal motor networks mediate the performance decline. Specifically, alterations in callosal connectivity between the primary motor cortex (M1) and the dorsal premotor cortex, as well as M1 and the dorsolateral prefrontal cortex mediate the bimanual performance decline during visuomotor tracking tasks in older adults (Serbruyns et al., 2015; Fujiyama et al., 2016), which suggests that the age-related performance decline is unlikely only due to difficulties in visual processing alone. RT decreased for all participants over the blocks and was generally slower for older participants and inverse-asymmetric movements, but no interaction between age and task or block could be observed. We decided to separate our performance measures into reaction time (RT) and accuracy (error) as the literature shows that older adults tend to favor accuracy over speed (Salthouse, 1979) and we were interested in whether changes in learning could be assigned to one of these domains. One methodological caveat for the reaction time data is, however, that a steady onset of the pinch movement could only be calculated by averaging over all repetitions of a condition in a block and hence we do not have the time course of RT improvements within a block. However, our data suggest that the accuracy in older adults is more sensitive to changes in task complexity than reaction time, this was even true when accuracy was calculated, like RT, based on individual averaged values for each condition and block. The finding that accuracy is more affected than speed is surprising as studies where the error is categorical, instead of continuous overwhelmingly suggest that older participants attempt to minimize errors even if that comes at the cost of slower reaction times (Salthouse, 1979; Baron and Mattila, 1989; Starns and Ratcliff, 2010). One potential explanation for the divergent results of our study is that speed accuracy calculations follow a different trajectory during a continuous tracking task than during a task with categorical errors.

### Associations between learning rate and motor-cognitive markers

Several motor-cognitive markers were associated with early bimanual learning in older and younger adults. Hand grip strength and few errors on the Cantab test battery were associated with higher learning rates while controlling for the initial performance and age group. This indicates that physical strength and cognitive–motor abilities are related to manual skill learning irrespective of age, and it is worth noting that similar associations have also been observed in children (Fernandes et al., 2016). The total learning rate over all three blocks on the other hand primarily predicted by a large initial error and did not show the same association with either strength or cognitive–motor ability. The finding that early learning can be associated with general cognitive and motor abilities is also in line with other studies, suggesting that the earliest phases of visuomotor learning engage most cognitive resources (Anguera et al., 2010). While our results are in line with previous studies suggesting the specific importance of grip strength and cognitive ability for motor learning, we could not confirm that an approximate measure of physical fitness such as the sit-to-stand test showed an association with learning (Niemann et al., 2016; Hübner et al., 2019).

## Conclusion

In this study, we observed that older adults have a different time course of early bimanual skill acquisition compared to younger adults, even if their net improvement over the whole training is comparable. We also observed that older adults were more challenged during complex bimanual movements, but task complexity did not interact with learning for either group. Finally, we observed that there is a positive association between motor–cognitive abilities, grip strength, and early learning rate in younger and older adults even when controlling for the initial task performance, which indicates physical and cognitive traits influence how quickly participants get better at a novel motor task.

## Data availability statement

The raw data supporting the conclusions of this article will be made available by the authors, without undue reservation.

## Ethics statement

The studies involving humans were approved by De Videnskabsetiske Komitéer-Region H; Journal-nr: H-21025010. The studies were conducted in accordance with the local legislation and institutional requirements. The participants provided their written informed consent to participate in this study.

## Author contributions

AZ: Data curation, Formal analysis, Investigation, Visualization, Writing – original draft, Writing – review & editing. KA: Conceptualization, Investigation, Methodology, Software, Writing – review & editing. AP: Formal analysis, Visualization, Writing – review & editing. MN: Conceptualization, Data curation, Software, Writing – review & editing. HS: Conceptualization, Writing – review & editing. JL-J: Conceptualization, Resources, Writing – review & editing. AK: Conceptualization, Formal analysis, Funding acquisition, Methodology, Project administration, Resources, Supervision, Visualization, Writing – original draft, Writing – review & editing.
